# Coumarins and Coumarin-Related Compounds in Pharmacotherapy of Cancer

**DOI:** 10.3390/cancers12071959

**Published:** 2020-07-19

**Authors:** Esra Küpeli Akkol, Yasin Genç, Büşra Karpuz, Eduardo Sobarzo-Sánchez, Raffaele Capasso

**Affiliations:** 1 Department of Pharmacognosy, Faculty of Pharmacy, Gazi University, Etiler 06330, Ankara, Turkey; busra.karpuz@gazi.edu.tr; 2 Department of Pharmacognosy, Faculty of Pharmacy, Hacettepe University, Sıhhiye 06100, Ankara, Turkey; yasin.genc@hacettepe.edu.tr; 3 Instituto de Investigación e Innovación en Salud, Facultad de Ciencias de la Salud, Universidad Central de Chile, 8330507 Santiago, Chile; eduardo.sobarzo@ucentral.cl; 4 Department of Organic Chemistry, Faculty of Pharmacy, University of Santiago de Compostela, 15782 Santiago de Compostela, Spain; 5 Department of Agricultural Sciences, University of Naples Federico II, 80055 Portici (Naples), Italy

**Keywords:** Benzopyrone, coumarin, cancer, drug discovery, natural product

## Abstract

Cancer is one of the most common causes of disease-related deaths worldwide. Despite the discovery of many chemotherapeutic drugs that inhibit uncontrolled cell division processes for the treatment of various cancers, serious side effects of these drugs are a crucial disadvantage. In addition, multi-drug resistance is another important problem in anticancer treatment. Due to problems such as cytotoxicity and drug resistance, many investigations are being conducted to discover and develop effective anticancer drugs. In recent years, researchers have focused on the anticancer activity coumarins, due to their high biological activity and low toxicity. Coumarins are commonly used in the treatment of prostate cancer, renal cell carcinoma and leukemia, and they also have the ability to counteract the side effects caused by radiotherapy. Both natural and synthetic coumarin derivatives draw attention due to their photochemotherapy and therapeutic applications in cancer. In this review, a compilation of various research reports on coumarins with anticancer activity and investigation and a review of structure-activity relationship studies on coumarin core are presented. Determination of important structural features around the coumarin core may help researchers to design and develop new analogues with a strong anticancer effect and reduce the potential side effects of existing therapeutics.

## 1. Introduction

Coumarins are polyphenolic compounds belonging a group of colorless and crystalline oxygenated heterocyclic compounds first isolated from the plant named *Dipteryx odorata* Willd. (Fabaceae) known locally as “coumaroun” by Vogel in 1820 [1,2]. Oxygenated heterocyclic compounds are furan derivatives with 4C atoms or pyran derivatives with 5C atoms. Although furan derivatives are rarely present in plants, pyran derivatives forming the structure of various compounds are encountered more frequently. The pyran derivatives are ketonic compounds that in the form of α-pyron or γ-pyron. Secondary metabolites called benzo-α-pyrone (coumarin) and benzo-γ-pyrone (chromone) occur due to condensation of pyron derivatives with benzene in plants [3,4].

Coumarin (1,2-benzopyrone or 2H-1-benzopyran-2-one) and coumarin derivatives are natural compounds that are widely available in plants as a heteroside or free form. A total of 800 coumarin derivative compounds that naturally found were obtained from about 600 genera of 100 families to date [5,6]. Coumarin and its derivatives are frequently found in the seeds, roots and leaves of many plant species belonging to families (especially Rutaceae and Apiaceae) in the Dicotyledonae class of the division of Spermatophyta. Although most natural coumarins are isolated from vascular plants, some coumarins such as novobiocin, coumermycin and aflatoxin are isolated from microbial sources [7,8].

These compounds have become of importance in recent years due to their various biological activities. Previous biological activity studies performed on coumarin derivatives revealed that these compounds have antitumor [9], photochemotherapy, anti-HIV [10,11], antibacterial and antifungal [12,13], anti-inflammatory [14,15,16], anticoagulant [inhibitors of the enzyme VKOR (vitamin K epoxide reductase)] [17,18], triglycerides lowering [19] and central nervous system stimulant effects [20]. However, a strong antioxidant and protective effect against oxidative stress by scavenging the reactive oxygen species has also been reported for hydroxycoumarins [21]. In addition, the discovery of coumarins with weak estrogenic activity has enabled the usage of this type of coumarins in the prevention of menopausal distress [22]. On the other hand, the usage of some coumarin derivatives as a tobacco flavor, which are used as fixative and flavoring agents, has been regulated by the FDA because of its negative effects, such as mild nausea, diarrhea and hepatotoxicity [23,24,25,26]. Besides their medical use, coumarins are also used in the cosmetic industry and agrochemical industry, as well as optical brightening agents [27,28].

Both natural and synthetic coumarin derivatives draw attention due to their photochemotherapy and therapeutic applications in cancer [29]. It has been reported that substitution patterns can affect the therapeutic, pharmacological and biochemical properties of coumarins in a positive way [18,22,30]. For instance, the substitution of a methoxy group at the 7-position and a 3-methyl 2-butenyl group at the 8-position of the osthol leads to a strong reduction of plasma alkaline transferase (ALT) level in hepatitis and inhibition of caspase-3 activation [31]. Some coumarins have cytostatic effect, while others have cytotoxic activity [32]. It has been revealed to show cytostatic activity of coumarin and its active metabolite, 7-hydroxycoumarin, on human cancer cell lines such as HL-60 (leukemia), MCF-7 (breast), A549 and H727 (lung) and ACHN (kidney). Moreover, cytostatic activity of these compounds against prostate cancer, malignant melanoma and metastatic kidney cell carcinoma has also been reported in clinical studies [32,33,34,35]. Compounds of 3 and 4-hydroxycoumarin structure were determined to inhibit cell proliferation in the gastric carcinoma cell line [36]. In vitro proliferation analysis investigating the mechanism of action of coumarins on the growth and metabolism of MCF-7 and A549 human tumor cells revealed that coumarin was not responsible for observed in vivo effects, but was a precursor of other active metabolites [4]. Previous studies showed that ortho- or meta-dihydroxycoumarins have more cytotoxic effect on human tumor cell lines than mono-hydroxycoumarins [37,38].

In the current review, compilation of various research reports on natural and synthetic coumarin derivatives with anticancer activity and investigation and review of structure–activity relationship studies on coumarin core were aimed. Determination of important structural features around the coumarin core may help researchers to design and develop new analogues with a strong anticancer effect and reduce the potential side effects of existing therapeutics.

### 1.1. Occurence

Coumarins are classified in four groups: simple coumarins, furanocoumarines, pyranocoumarins and pyrone-substituted coumarins [5].

**Figure 1:**
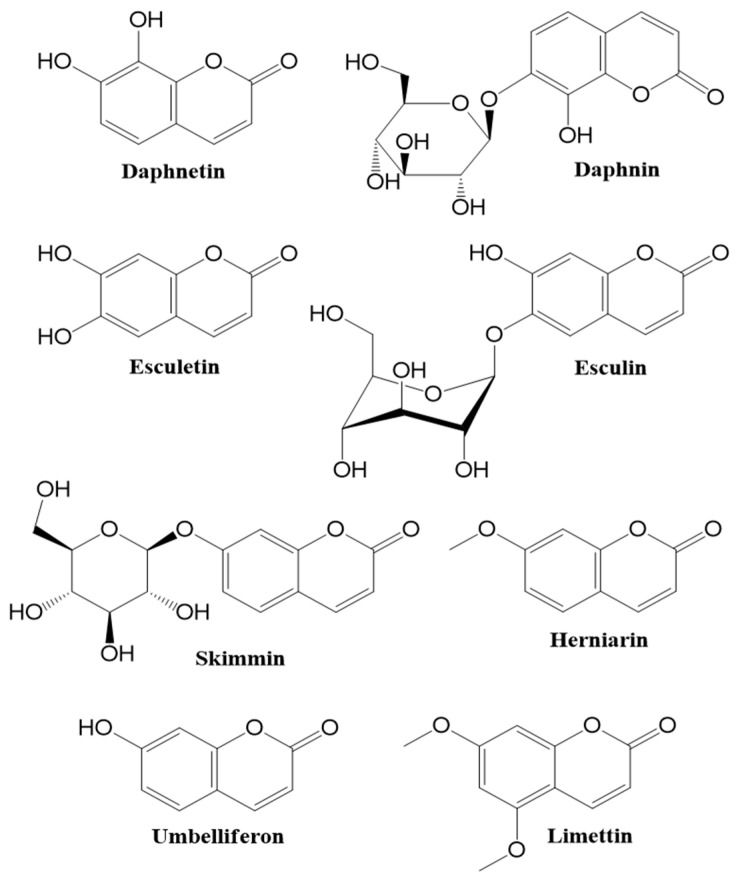
Chemical structures of some simple coumarins.

Simple coumarins: these are composed of hydroxylated, alkoxylated and alkylated derivatives of coumarin and their glycosides (e.g., Umbelliferone, skimmin, limettin, herniarin, esculetin, esculin, daphnetin and daphnin (Figure 1)) [5]. 

**Figure 2:**
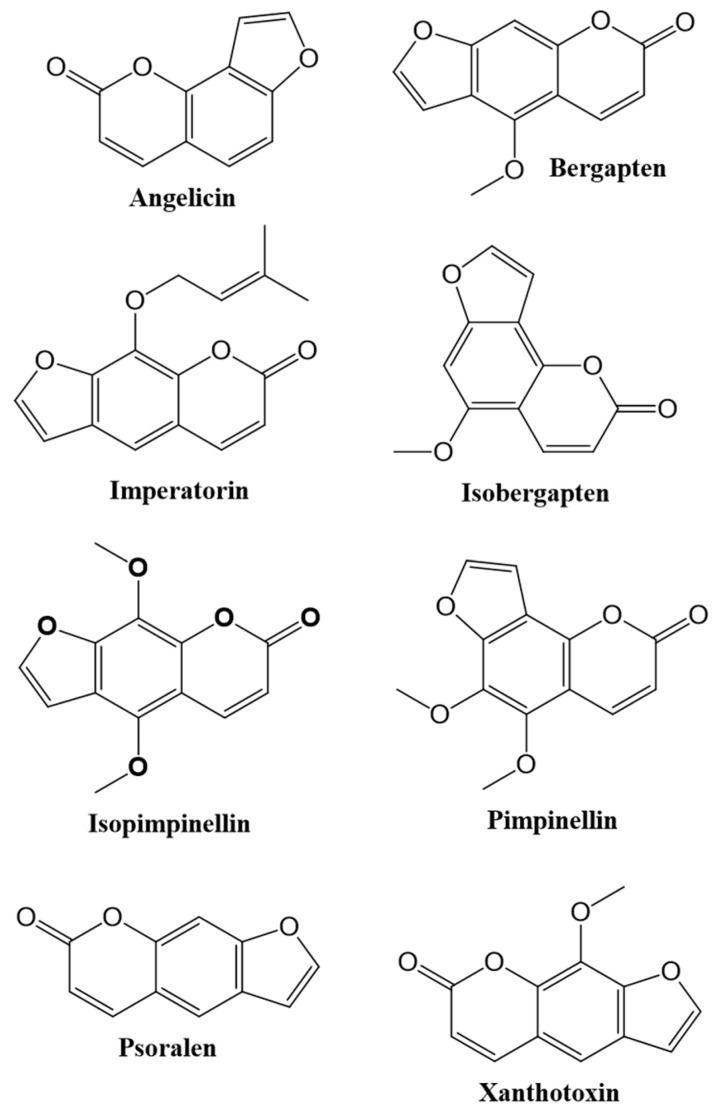
Chemical structures of some furanocoumarins.

Furanocoumarins: This group of coumarins consists of a furan ring fused with a coumarin. They are divided into two groups as C6/C7 (linear) type, C7/C8 (angular) type according to the attachment place of the furan ring. (e.g., psoralen, xanthotoxin, bergapten, imperatorin, isopimpinellin, anjelisin, isobergapten and pimpinellin (Figure 2)) [3,5,39,40,41,42].

**Figure 3:**
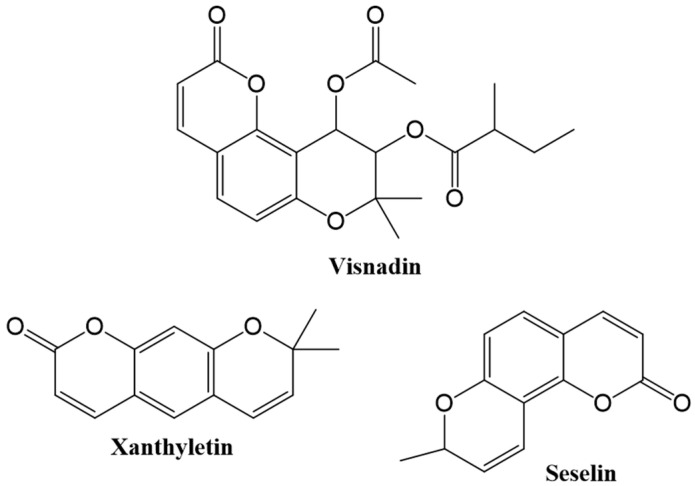
Chemical structures of some pyranocoumarins.

Pyranocoumarins: six-membered pyran ring is fused with the benzene ring via C6-7 (linear) or C7–8 (angular) (e.g., visnadin, xanthyletin and seselin) (Figure 3) [4,43].

**Figure 4:**
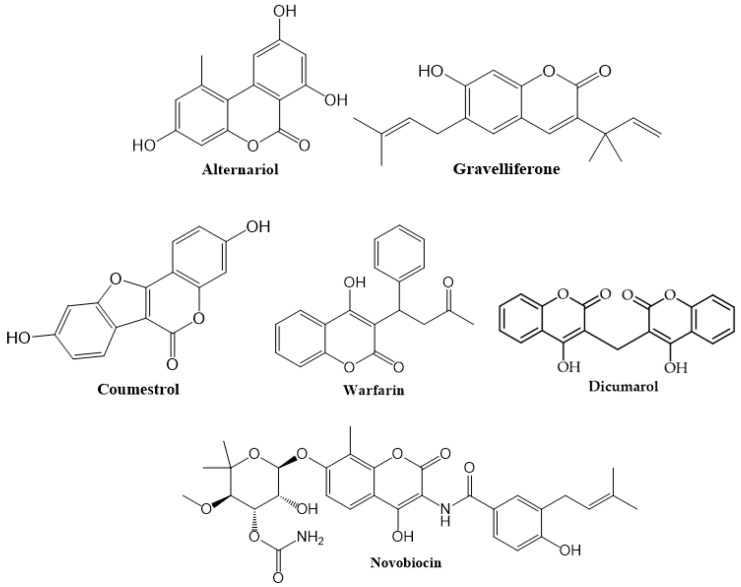
Chemical structures of some pyrone-substituted coumarins.

Pyrone-substituted coumarins: These are classified in three groups: 4-Hydroxycoumarin (Novobiocin and Dicumarol), 3-Phenylcoumarin (Coumestroln and Gravelliferone) and 3,4-Benzocoumarin (Aeternaryiol). 4-Hydroxycoumarins are not found in plants in free form. Warfarin, a synthetic compound, belongs to this group (Figure 4) [39,44]. 

Multi-target compounds are recently being searched for and these compounds are thought to be promising compounds for the treatment of several disorders, including cancer and heart failure. In this context, compounds observed from natural sources come into prominence due to their low toxicity, low drug resistance, low cost and high efficacy [45,46]. Therefore, new compounds isolated from natural sources, such as plants and animals, and possible combination of these compounds with conventional chemotherapeutic agents seems to be important strategies to improve life quality, especially in cancer patients [47]. Coumarin-based structure compounds constitute a major group of natural compounds with various pharmacological effects. These group of compounds can be isolated from different plants, including Achillea, Artemisia and Fraxinus genera, and also, they can be synthesized through various chemical reactions. Several strategies such as maceration, reflux, ultrasonic-assisted and microwave extraction methods are used for the isolation and purification of coumarin compounds. Perkin, Von Pechmann, Knoevenagel and Wittig organic reactions are some of reactions that coumarins can be synthesized [46]. In the biosynthetic origin of coumarins, the shikimic acid pathway plays an important role. In this pathway, there are several enzymatic steps leading to occur chorismic acid, cinnamic acid, p-coumaric acid and umbelliferone. Moreover, the cytochrome P450 enzymes have a crucial role in the ortho-hydroxylation of cinnamic acid leading to occur umbelliferone, scopoletin and isofraxidin [46,48]. 

### 1.2. Cancer

Cancer, known as the abnormal division, proliferation and accumulation of cells in an organism, is one of the most common causes of disease-related deaths worldwide. It can affect a single organ as well as spread to distant organs [27].

Since the division and growth of cells are controlled by genes, cancer is basically a disease associated with genes. The genes on the chromosomes are tightly packed, and physical or chemical changes on these genes can directly affect the cell’s function. Although DNA repair systems can ameliorate the function of the gene in case of damage, they cannot be always successful. In this case, inadequate or incorrect production of proteins as the products of genes leads to the disruption in cellular functions. Another factor that affects the function of the gene is epigenetic modifications, such as methylation, acetylation, phosphorylation and ribosylation, which change the function of the gene without changing its structure. These modifications can only act on a specific site, but also, they may cause of regional deletions, insertions or inversions that affect all or a large part of the chromosomes [27,49,50]. 

**Figure 5:**
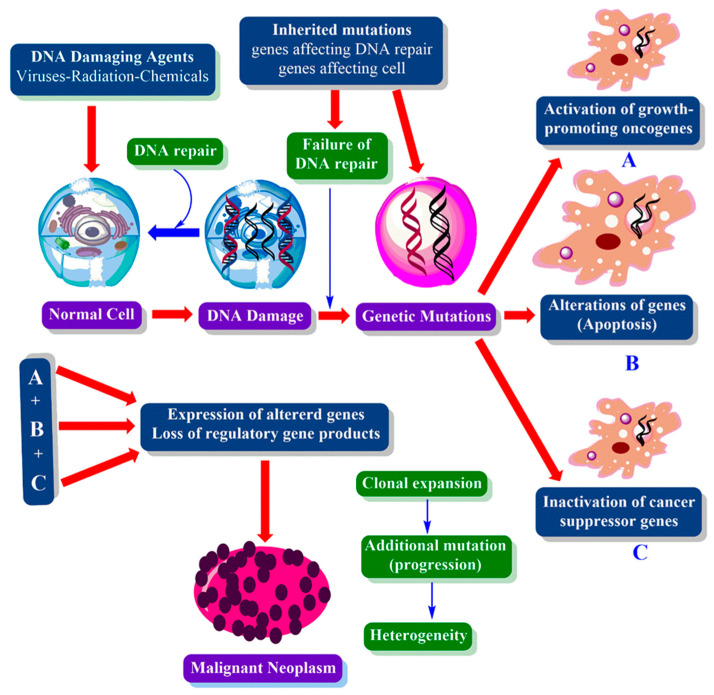
Schematic representation of cancer mechanism involving oncogenes (genes such as RAS, Erk and MYC), tumor suppressor genes (TP53 gene) and DNA repair genes.

There are three gene groups that play crucial role in cancer formation: oncogenes (genes such as RAS, Erk and MYC), tumor suppressor genes (TP53 gene) and DNA repair genes. Proto-oncogenes, which are normal genes that enable cell growth and differentiation, can be active and turn into oncogenes due to mutations, increased gene expression, gene duplications and/or chromosomal rearrangements. Tumor suppressor genes control cell division and proliferation, initiate DNA repair in case of damage and trigger apoptosis if repair attempt falls down. Deletions, point mutations, epigenetic gen silencing, improper separation of chromosomes and mitotic recombinations can lead to loss of control of the tumor suppressor gene, resulting in loss of control in the cell cycle and carcinogenesis. Another important group of genes are DNA repair genes that attract the necessary proteins to the site of damaged DNA, thereby restoring the function of the gene. Providing the destroying of cell’s apoptotic or necrotic pathway in case of ineffective repair is another important function of DNA repair genes. However, loss of function in this important gene group is a common problem in cancer formation of the cell. One of the most known DNA repair genes is the breast cancer (BRCA) gene, which causes breast cancer due to impaired function [49,50] (Figure 5).

**Figure 6:**
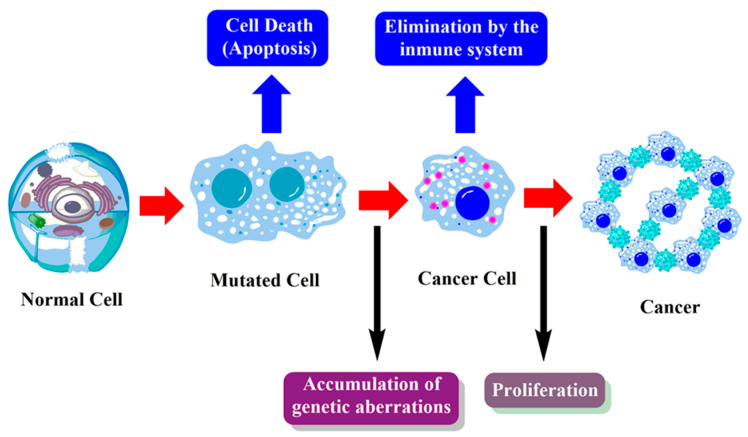
Basic mechanism of carcinogenesis.

Under normal conditions, when cells receive signals from the outer membrane, they grow, divide and proliferate. The signals coming from outer membrane enter into the cell, transferred to the nucleus and the process begins. Before the division, a cell checks its surroundings and checks whether there is enough nutrients and place to grow, and begins to grow if conditions are favorable. They grow until they reach the predetermined size and number and stop growing as they touch each other (contact inhibition or contact-related growth stop). In case of damage of one of the DNA or cell elements, the cells stop growing and dividing in order to move to a phase called the G0 phase which provides repairment. If the cell is repaired with the necessary arrangements in this phase, it is included in the circulation again and continues its life. However, if a cell is damaged beyond repair, apoptosis is initiated resulting in death of the cell or the immune system cells demolish the damaged cell. Thus, transferring the damaged DNA to the next generations is prevented. On the other hand, cancer cells have their own signal systems that allow abnormal growth differently from normal cells, and do not stop dividing after contact with other cells; they continue to grow and proliferate. They can form new vascular systems (neo-vascularization) by affecting the stroma around them in order to get the necessary nutrients and oxygen. They can continue eternal replication by fixing their telomeres or maintaining telomerase activity [49,50]. They can enter the circulatory system and move to different organs, causing metastasis. They can evade apoptosis and are not genetically/epigenetically stable (Figure 6).

Tobacco and tobacco products, alcohol, malnutrition, obesity, viruses, exposure to ionizing rays, occupational diseases and environmental pollutants can cause cancer [49]. Lung cancer is the most common type of cancer that causes death in both men and women. In the second place are prostate cancer in men and breast cancer in women [50].

### 1.3. Cancer Treatment

Although some standards have been determined for cancer therapy, various approaches and treatments are applied for specific to each type of cancer. Biological therapies, such as radiotherapy, chemotherapy, surgery, immunotherapy, hormone therapy, targeted therapies and gene therapy can be used alone or in combination in cancer therapy [51,52]. However, these methods, known as the gold standard, have advantages as well as disadvantages. Despite the discovery of many chemotherapeutic drugs (Adriamycin, Cisplatin, Campotins, Vinblastin, Mercaptopurine, etc.) that inhibit uncontrolled cell division process for the treatment of different types of cancer [53,54], serious side effects of these drugs on hematopoietic system, bone marrow and gastrointestinal epithelial cells and hair follicles are a crucial disadvantage [55,56,57,58]. In addition, multi-drug resistance (MDR) is another important problem in anticancer treatment [59]. Due to problems such as cytotoxicity and drug resistance in existing chemotherapeutic agents, many investigations are being conducted to discover and develop effective anticancer drugs. Previous studies showed that many compounds obtained from natural resources can be used as preventive and therapeutic agents in cancer therapy. These compounds have been shown to increase the effectiveness and tolerance of chemotherapeutic agents when used in combination with chemotherapy or alone in various types of cancers [60,61,62,63,64,65,66,67]. In recent years, researchers have focused on the anticancer activity of coumarin and coumarin-derived natural compounds among the large number of phytochemicals, due to their high biological activity and low toxicity. Coumarins are commonly used especially in the treatment of prostate cancer, renal cell carcinoma and leukemia [54,68,69], and they also have the ability to counteract the side effects caused by radiotherapy [36,38,70,71,72]. Mahler et al. revealed that combination of coumarin and troxerutin have a positive effect on the treatment of malignity of head and neck radiotherapy [73]. Both coumarin and coumarin derivatives are promising compounds as potential inhibitors of cell proliferation in various carcinoma cell lines [8,74].

## 2. Roles of Coumarins in Anticancer Activity 

Studies conducted on the anticancer activity of coumarin and its derivatives revealed that the mechanism of action of these compounds is generally caspase dependent apoptosis.

CYP 2A6, an isoform of cytochrome P450, metabolizes coumarin to 7-hydroxycoumarin, which has an antiproliferative effect by reducing Bcl expression in various organs and tissues. Bcl-2, a 26 kDa membrane protein, blocks free oxygen radicals, inhibits mitochondrial CYP and suppresses activation of caspase-9, which extends the cell life cycle cumulatively [75]. Thus, it causes carcinogenesis and leads to accumulation of oncogenic mutations in the normal cell. Caspase-9 is activated by Bax, a membrane protein. Over-expression of Bax causes mitochondrial cytochrome C to be released into the cytoplasm through modulation in the mitochondrial membrane. Cytochrome C in the cytoplasm activates caspase-9 and activation of caspase-9 leads to the caspase-3, 6 and 7 activation which breaks down key cytoplasmic and nuclear proteins [76,77].

Coumarins regulate the fate of normal cells by modulating signal transduction pathways containing GTP-binding proteins and reducing Bcl-2 expression. The Bcl-2 protein family consists of Group 1 (Bcl-2 and Bcl-XL), which are apoptotic, and Group 2 (Bax, Bad, Bid and Bak), which are pro-apoptotic. BH1, BH2 and BH3 in the Bcl-2 protein family and their dimerization determine the sensitivity of a cell to negative stimuli [78]. As the ERK/MAPK pathway actively participates in cell proliferation and cytokine production, this pathway is used as an important target for the development of new anticancer agents. This pathway is regulated by MEK1/2 which is activated by direct phosphorylation through MAP3Ks such as ERK1/2, RAFs (a-Raf, b-Raf and c-Raf), COT and MOS [79,80]. Coumarin and its derivatives are one of the highly specific allosteric inhibitors of MEK1/2. MAP3Ks also inhibit the activation of MEK1/2 by inhibiting upstream modulation. However, the activity of activated MEK1/2 is not affected by these compounds [81,82].

Inhibition of the Hsp90 protein is another mechanism in cancer treatment. Novobiocin (Nvb) and other analogs contain coumarin moiety interact with an ATP-linked molecular chaperone that modulates the folding of many proteins, including kinases and transcription factors which are directly related to cancer. Novobiocin and its analogs inhibit the Hsp90 via causing the degradation of Hsp90 proteins by the ubiquitin proteasome pathway.

Cdc25 phosphatases are enzymes that control the eukaryotic cell cycle [83]. Cdk/cyclin are activated by Cdc25A, Cdc25B and Cdc25C enzymes, which are dual specificity phosphatases in normal cell cycle in humans, via the dephosphorylation on pThr14 and/or pTyr15 residues [84]. Cdc25A, Cdc25B and Cdc25C enzymes are involved in the control of the G2/M phase of the cell cycle while the G1/S phase is controlled by Cdc25A [85]. The genomic stability of the living system is maintained by strict regulation of transitions between each of these cell cycle phases. The hyperactivity of Cdc25 phosphatases disrupts this genomic stability, leading to uncontrolled cell growth [84]. These enzymes control and direct each state of cell division. Therefore, they are known as central regulators of the cell cycle [86]. Increased expression of Cdc25A and Cdc25B phosphatases causes negative prognosis in cancer [87]. Many coumarin derivatives, which are potent inhibitors of Cdc25 phosphatase, can be used to control different tumors.

p53 is a transcription factor that stops cell growth and plays an important role in apoptosis. However, apoptosis can also be activated by binding of the target genes to the DNA sequence and activation of p21 and Bax, the promoters of the subgenes. 7,8-Dihydroxy-4-methylcoumarin induces apoptosis by down-regulating p53, Bax, p21 and COX-2, up-regulating c-Myc protein and reducing ERK1/2.

**Figure 7:**
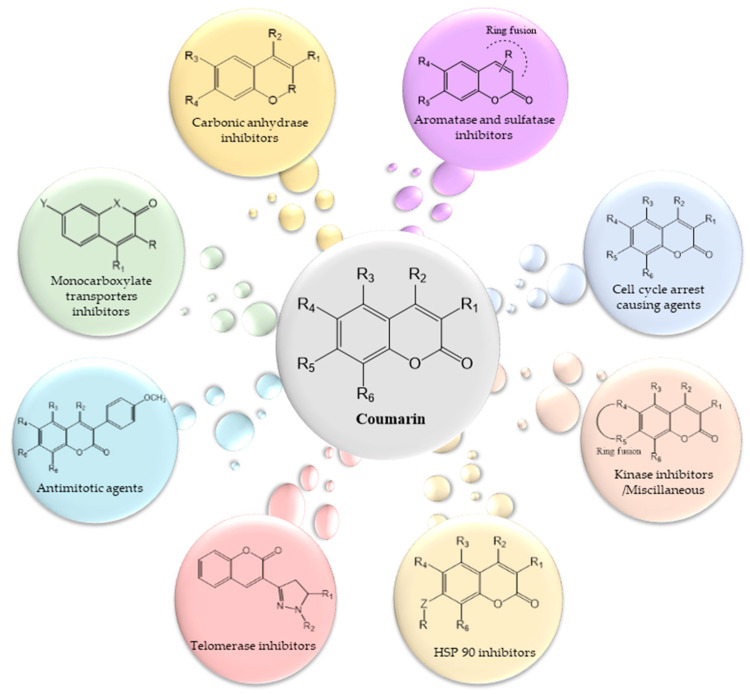
Roles of coumarins in anticancer activity with structure activity relationship.

The general structure activity relationship (SAR) and anti-cancer activity of coumarins are presented in Figure 7. 

A coumarin compound, esculetin, exhibits many pharmacological effects related with cell proliferation and so antitumor efficacy. Polylactide-co-glycolide (PLGA) nano-micelles formulation of esculetin, nano-esculetin, was prepared to treat against insulinoma INS-1 cells which release more insulin than normal beta cells. Results of this study revealed that administration of nano-esculetin decreased the cell viability more significantly than free esculetin in vitro. Free esculetin also decreased the viability of cells in vitro; however, it has been known that nano-formulations exhibit their superior efficacy at in vivo conditions due to enhanced permeability and retention (EPR) effect. Therefore, nano-formulation of esculetin is thought to be more effective than free esculetin at in vivo conditions [88].

### 2.1. Coumarins in Breast Cancer

The breast tissue is composed of lobules formed by the glands that produce milk, ducts that allow milk to be discharged and fat and connective tissues. Lobes are formed by combination of the lobules, and each breast has 15–20 lobes. The lobules are connected to each other by milk ducts and milk ducts join towards the nipple. The development and physiological functions of the breast are regulated by hormones. The main hormones that provide the development of breast tissue are estrogen and progesterone.

Breast cancer is a systemic disease that occurs when the cells lining the mammary glands and milk ducts proliferate abnormally, spread to various tissues and organs and continue to grow there. It is a complex disease that affects women physically, psychologically and socially [89], and ranks first among cancer types seen in women in the world, and also second most common cause of death due to cancer following the lung cancer [50,90]. In epidemiological studies, the prevalence was found to be 22–26% and the risk of breast cancer-related mortality was around 18% [91,92]. Risk factors related to breast cancer development are summarized in Table 1.

Since the breast consists of two main structures, there are two types of breast cancer: lobular cancer developing from the milk secreting part and ductal cancer developing from the milk ducts. The most common type of breast cancer is ductal cancer and accounts for 75% of all breast cancers. Breast cancers are histologically divided into two main groups, in situ and invasive carcinomas. In in situ carcinoma, malign epithelial cells are limited in ductus and acinus surrounded by basement membrane while in invasive (infiltrative) carcinoma, neoplastic cells cross the basement membrane and show invasion to the stroma. While the malignant breast tumors have been classified traditionally according to their histological appearance, today some subtypes have been defined according to their molecular features [93,94,95,96]. The subtypes of breast cancers have been identified according to the presence of estrogen receptor (ER) in the light of gene expression studies by Perou et al. for the first time [97]. According to this valid classification, ER positive tumors contain gene expression similar to luminal cells of the mammary glands, cytokeratin profile and markers associated with other luminal cells. In contrast, some of the ER negative tumors are immunohistochemically positive for human epidermal growth factor receptor-2, cerb-B2 (HER2) or HER2 gene amplification may be demonstrated in these tumor cells. This group is known as HER2 positive tumors. HER2 negative luminal non-group tumors show gene expression and immune reactivity similar to normal basal cells of mammary glands. Since ER and progesterone receptor (PR) are also negative in this type of tumors, this group is called basal-like or triple negative tumor group [97,98,99,100,101]. As a result of studies and meta-analyzes, it was determined that 75% of breast tumors ER and/or PR positive, that is, most tumors are in the luminal group [95]. However, tumors in the luminal group are divided into subtypes as luminal A and B because of their different behaviors. Tumors of luminal A group, which has the highest prevalence among breast cancers, consist of HER2 negative tumors with low proliferative activity, mitotic rate and histological grade. The prognosis of patients with luminal A tumor is good and metastases are often limited to bones. Luminal B tumors are more malignant and the most important difference of this group is that tumors have high proliferation rate. The limit value between luminal A and B is generally considered to be nuclear Ki67 expression immunohistochemically less than 14% of tumor cells. In addition, approximately 30% of HER2 positive tumors are immunohistochemically in the luminal B phenotype [102,103,104,105,106,107,108,109]. 

Despite the development of early diagnosis strategies and advances in treatments, breast cancer is still an important reason of mortality and morbidity. Prognostic factors known in breast cancer are lymph node involvement, tumor size, distant metastasis status, tumor cellular differentiation degree, patient’s age, state of hormone receptors in tumor, HER2 overexpression, tumor proliferation index, lymphovascular invasion, tumor histology, response to neoadjuvant chemotherapy and hormonotherapy and p53 mutation. 

In premenopausal women, high levels of androstenedione compete with aromatase inhibitors in the enzyme complex in cases which estrogen synthesis cannot be completely blocked, and an initial lowering of the estrogen level causes an increase in the level of gonadotropin. The main source of estrogen in postmenopausal women is the conversion of androstenedione released from the adrenal gland into estrogen through the aromatase enzyme in the peripheral tissues [110]. The aromatase inhibitors (AI) used at this stage lower the plasma estrogen level by inactivating or inhibiting the aromatase [111]. The presence of hormone-induced tumors, including stimulation of the estrogen receptor, has been reported in about one-third of postmenopausal breast cancer patients [112]. In recent preclinical and clinical studies, the synthesis of estrogen receptor agonists/antagonists has gained importance in the prevention and treatment of breast cancer [113]. ER antagonists are commonly used in the treatment of postmenopausal women and hormone-induced breast tumors. Aromatase and sulfatase pathways play a role in the synthesis of estrogens formed only in peripheral tissues. The aromatase pathway ensures that the androgen precursor androstenedione, which is mainly secreted by the adrenal cortex, is converted into estrogen by the aromatase (AR) enzyme complex, while the estrone sulfatase pathway (E1-STS) provides the conversion of the aromatase-induced estrone to estrone sulfate (E1S) by sulfotransferase enzymes [114]. In breast tumors, the activity of the sulfatase enzyme is higher and leads to poor prognosis [115,116,117,118]. The E1-STS pathway is considered the main source of estrogen formation, which causes a fairly strong response in patients with ER + breast tumors [119,120,121]. This approach led to the discovery of new coumarins as STS [122,123,124,125] and AR inhibitors [126].

AI provides lowering in the level of estrogen and thus prevents breast cancer by reducing cell proliferation, which includes the inhibition of the formation of genotoxic metabolites of estrogen. Genotoxic estrogen metabolites are (i) catechol estrogens that covalently bind to DNA and induce mutations that initiate cancer; (ii) 2-hydroxyl-estradiol forming stable DNA insert; and (iii) 4-hydroxy-estradiol, a potential carcinogenic metabolite that causes 8-hydroxylation of guanine bases leading to estrogen induced indirect DNA damage [127,128,129].

Aromatase inhibitors are the standard option in postmenopausal breast cancer [130]. Previous studies have revealed that benzopyranone substrates, such as 4-benzyl-3-(4’-chlorophenyl)-7-methoxy-coumarin, are stronger competitive AI than aminoglutethimide. It has been reported that the specific interaction of this compound with AR shows a greater decrease in binding to the active site of AR and suppressed the proliferation of AR and ER positive MCF-7 breast cancer cells [126,131,132].

There is an over-expressed ER in the breast tumor cell at an early stage of cancer and during hormonal therapy [133,134]. Many antitumor agents used in treatment have non-selective effect and acute toxicity so use of these agents in the treatment is limited [135]. Conjugation of cytotoxic drug components to a carrier with selective activity to tumor tissues is an effective strategy in the development of effective antitumor drugs with a high therapeutic index [136,137,138,139,140,141]. Studies have shown that combining the cytotoxic agent with steroid hormones provides target selectivity of the conjugate and allow conjugates to accumulate in ER-rich cells as a result of improving antitumor activity and binding to ER [142,143,144,145,146]. 

In a study investigating the antiproliferative efficacy of new bioconjugates containing 3-substituted coumarins and estradiol, highly antiproliferative activity of compounds on noninvasive and invasive breast cancer cell lines (MDA-MB-231/ATCC and NCI/ADR-RES MDA-MB-435) has been revealed [147].

Cui et al. showed the anticancer effects of three synthesized coumarins derived from triphenylethylene (TCHs), occurring through the inhibition of angiogenesis on breast cancer cell lines. Compound TCH-5c inhibited proliferation, resulted in cell death, increased p21 protein expression to induce G0/G1 arrest and changed endothelial cell cytoskeleton organization and migration in EA.hy926 endothelial cells. In addition, this compound inhibited breast cancer cell line derived VEGF secretion, decreased breast cancer cell-induced endothelial cell tube formation in vitro and suppressed SK-BR-3 breast cancer cell-initiated tumor formation in vivo. These results have potential implications in developing new approaches against breast cancer [148].

### 2.2. Coumarins in Leukemia

Leukemia is a clonal disease that results from neoplastic exchange of hematopoietic precursor cells in the bone marrow. Marrow damage occurs when a large number of immature and malignant cells replace normal marrow cells. Thus, decrease begins in the number of platelets involved in blood coagulation and the number of leukocytes involved in defense. This causes intense injuries and bleeding in patients with leukemia, as well as easy infection. Moreover, the defense mechanism weakens and may cause anemia and shortness of breath in advanced stages. Leukemia has symptoms such as weakness and fatigue, fever, some neurological symptoms, bloating and bleeding in the gums.

Leukemia is a type of cancer which effects the blood production system (lymphatic system and bone marrow) in the body. Leukemia is classified as acute or chronic (they are subdivided according to their appearance under the microscope) and according to the spread and development characteristics of the tumor. Generally, acute leukemia occurs in children, while chronic leukemia tends to be more common in adults. There are different types of blood cancers according to the cell type (such as myeloid and lymphoid) and the duration of the disease. Some types of blood cancers show a faster and poor prognosis. Leukemia is more common in childhood than other types of cancers, and 30–35% of cancers in this period are composed of leukemia. Frequency is 3–4 in 100,000 in children under 15 years of age in western countries.

Although the causes are not known exactly, both genetic and environmental factors are thought to play an important role in leukemia. Mutations in DNA in somatic cells cause activation of oncogenes or inactivation of tumor suppressor genes. Thus, regulation of cell death and division is damaged. Apart from genetic reasons, this damage is thought to be caused by petrochemicals, radiation, carcinogens and some viruses (e.g., HIV).

The purpose of leukemia treatment is to reduce the number of white blood cells. Since surgery cannot be performed for this purpose, the basis of treatment is the use of cancer drugs, the combination of chemotherapy and radiotherapy, and bone marrow transplantation in cases where chemotherapy is insufficient.

Egan et al. have reported that 8-nitro-7-hydroxycoumarin showed cytotoxic properties and induced apoptosis in the tested HL-60 and K562 human leukemia cell lines [74]. In previous studies investigating the effects of coumarin, 6-hydroxycoumarin, 7-hydroxycoumarin and esculetin on the growth, metabolism and cell signal of human tumor cell lines, it was determined that esculetin has the strongest antiproliferative effect on the tested carcinoma cell lines [8,149]. In a study by Cooke and O ’Kennedy, it has been reported that 7-hydroxycoumarin and esculetin inhibit tyrosine phosphorylation in EGF-stimulated tumor cells in a time and dose-dependent manner, and the effect can be achieved by reducing tyrosine kinase activity of the EGF receptor [8].

Esculetin was found to inhibit cell growth and cell cycle progression by inducing G1 phase retention in human leukemia HL-60 cells in the study by Wang et al. In this study, it has been shown that esculetin provides a significant increase in the level of hypophosphorylated retinoblastoma protein (pRb) and a decrease in CDK4 level, and also significantly upregulation of p27 and downregulation of cyclin D1. The results suggested that, as a result of inhibited pRb phosphorylation, esculetin can inhibit the growth of human leukemia HL-60 cells by stopping the G1 phase cell cycle [150].

In the study conducted by Jimenez-Orozco et al., 7-hydroxycoumarin was found to have more cytostatic activity than coumarin on the human adenocarcinoma cell line A427, which has pRB positive and homozygous deletions in the p16INK4a gene. However, it has been reported that inhibition of cell cycle in G1/S transition is consistent with the cytostatic effect of 7-hydroxycoumarin and does not create an alteration in cyclin D1 mRNA level after transcription [151].

Taniguchi et al., reported that methoxyinophyllum P, calocoumarin B, and calophyllolide isolated from the leaves of *Rhizophora mucronata* showed cytotoxic effects against HL-60 cells (promyelocytic leukemia cells) with IC50 of 12.9, 2.6, and 2.2 µM [151]. Furthermore, armenin, hemiterpene ether coumarin, isolated from *Artemisia armeniaca* Lam, showed cytotoxic effects with IC50 equal to 22.5 and 71.1 µM for K562 and HL-60 cells (chronic myelogenous leukemia cells), respectively [152].

### 2.3. Coumarins in Malignant Melanoma

Although malignant melanoma (MM) is the third most common skin cancer, it is the skin cancer with the increasing incidence and highest mortality in the world. It is anticipated that 1 out of every 75 people born in 2000 will develop malignant melanoma in any period of their lives, and 20% of them will die due to common disease within 5 years after diagnosis [153,154]. MM accounts for 1.5% of all cancers and approximately 1% of cancer-related deaths. 40% develop from a previously existing nevus. Intermittent and intense exposure to sunlight is the biggest risk factor among environmental risk factors, especially when combined with endogenous factors (Fitzpatrick 1 and 2 skin types, immune deficiency syndromes and genetic predisposition) [155,156]. The risk of malignant melanoma increases 1000 times in patients with genetic anomalies such as xeroderma pigmentosum (XP) [157]. Risk factors related to malignant melanoma development are summarized in Table 2.

The purpose of the treatment of patients with malignant melanoma is to locally control the disease and to prevent lymph node and remote organ spread, if possible. Early diagnosis and adequate primary surgical resection form the basis of malignant melanoma treatment [158].

Warfarin, which is a coumarin derivative, may play an important role in the treatment of malignant melanoma due to its effects such as preventing tumor spread, stimulating granulocyte, lymphocyte and macrophages [159]. In the study conducted by Thornes et al., coumarin and warfarin have been reported to prevent recurrence of malignant melanoma as macrophage and dose dependent [34,160]. Velasco-Velazquez et al. revealed that 4-hydroxycoumarin disrupts the actin cytoskeleton in murine melanoma cell line B16-F10 but does not affect benign fibroblastic cell line B82 and may be useful as adjuvant therapy in melanoma as it prevents the adhesion of tumor cells to extracellular matrix during metastasis [161].

### 2.4. Coumarins in Renal Cell Carcinoma

Renal cell carcinoma (RCC) is responsible for approximately 3% of tumors seen in adulthood. In recent years, especially with the widespread use of imaging methods, there is an increase in the incidence of RCC at every stage and mortality rates resulting from disease [162]. One third of patients with RCC are metastatic at the time of diagnosis, or one third of them develop metastases despite treatment. Most of the tumors in RCC are associated with large, locally advanced and often lymph node, renal vein or vena cava invasion, and histologically, approximately 90% are transparent cell types [163]. Metastasis occurs in many organs, especially lung, liver, bone, adrenals, pancreas, brain, thyroid gland, skin and ureter in order of frequency. While the transparent cell type metastasizes to the lungs more often, papillary type makes it to the lymph nodes and the chromophobes to the liver [164,165]. The average survival in these patients is 10–12 months, and the 2-year survival rate is 18–20%. [166,167].

There have been promising developments in prognosis with the introduction of new molecules that make up immunotherapy and targeted therapy, a better understanding of the timing and effectiveness of cytoreductive nephrectomy, and the use of these methods together. Response rates do not exceed 10–15% in immunotherapy using IL-2 and interferon alfa [166]. Tyrosine kinase inhibitors (bevacizumab, sunitinib, sorafenib) used in targeted therapy act by vascular epithelial growth factor (VEGF) receptor blockade and mTOR inhibitors (temsirolimus and everolimus) by reducing regulatory factors that can be induced by hypoxia. These drugs are applied as first and second line treatment in metastatic renal cell carcinoma, resulting in significant improvements in total and disease-free survival times [168,169].

It has been reported in clinical studies on metastatic renal cell carcinoma patients that 14 of 45 RCC patients showed positive results with almost no toxic/side effects in case of use of coumarin at the oral dose of 100 mg/day and with the addition of cimetidine at the dose of 4 × 300 mgs/day from the 15th day of administration [170,171,172]. In dose and toxicity studies conducted by Marshall et al., it was determined that coumarin was well tolerated at the applied doses (600–5000 mgs) and it was thought that nausea, which was determined as a common side effect, was caused by the intense aroma of coumarin [173]. Finn et al. reported that 6-nitro-7-hydroxycoumarin and 7,8-dihydroxycoumarin showed irreversible cytotoxic effects in human renal carcinoma cells and non-carcinoma proximal tubular cells. However, it was determined that mentioned compounds were not mutagenic in the Ames test [68]. A derivative, consisting of 1,2,4-triazolin-3-one attached to 4-methylcoumarin, was found to have hopeful activity against RCC cell line [174]. A recent derivative, coufin, a novel indolylcoumarin, displayed potent anticancer activity both in 2D (monolayer culture) and 3D (tumor spheroid culture) by inhibiting microtubule formation and blocking the cell cycle at G2/M [175]. The results obtained suggest that the investigated coumarins may play a potential therapeutic role in the treatment of renal cell carcinoma.

### 2.5. Coumarins in Prostate Cancer

Prostate cancer is the most common solid tissue cancer that occurs with the uncontrolled growth of cells in the prostate gland. Cancer cells primarily grow uncontrolled and spread into the prostate. These then reach the capsule surrounding the prostate, pierces the capsule and spreads out of the prostate. Unlike benign prostate gland enlargement, prostate cancer does not originate from the center of the prostate, but from its decentralized, distal center. Therefore, urinary complaints in prostate cancer disturb the patient in the later period. It is characterized by a very slow growth rate and broad biological variability in terms of hormonal sensitivity [176,177]. It can spread to nearby organs, the lymphatic system and other parts of the body through the bloodstream during the period of growth and spread. Prostate cancer has a slow course, but the tumor may show a rather aggressive character and spread to the bones and other organs. According to the data of American Cancer Society, men have been reported to have 16.7% risk of development of prostate cancer life-long, and a 2.5% risk of life loss. One in every 5–6 men is at risk of developing prostate cancer throughout their life. When prostate cancer is diagnosed at an early stage, it is among the cancer types with high treatment success. While the treatment options of prostate cancer vary according to the growth rate and spread of cancer and general health status of the patient, the treatment options are surgery, chemotherapy, radiotherapy, hormonal therapy or their different combinations.

In phase I trial studies of 40 patients with metastatic, hormone-naive or hormone-refractory prostate cancer, positive results were obtained with 3 g of coumarin daily in patients with low tumor burden. However, during the 7 years following the study, it was found that prostate-specific antigen (PSA) levels remained stable, with only three bone metastases [35,72]. In the study of Myers et al., coumarin was found that after 5 days of treatment at concentrations of 0, 10, 50, 100, 250, 500 μg/mL, it inhibits the proliferation of two malignant prostate cell lines (DU145 and LNCaP) [29].

### 2.6. Coumarins and Other Cancers 

Taniguchi et al., reported that methoxyinophyllum P, calocoumarin B, and calophyllolide isolated from the leaves of *Rhizophora mucronata* displayed anticancer activity against HeLa cells (cervical cancer) with IC50 equal to 3.8, 29.9, and 36.4 µM [178].

Clausarin isolated from the root bark of *Clausena harmandiana*, showed high cytotoxic activity which was superior to that of cisplatin used as a positive control, against hepatocellular carcinoma (HepG2, IC50 = 17.6 ± 2.1 µM), colorectal carcinoma (HCT116, IC50 = 44.9 ± 1.4 µM) and lung adenocarcinoma (SK-LU-1, IC50 = 6.9 ± 1.6 µM) cell lines. From a mechanistic point of view, apoptosis induction was reported as an anticancer mechanism of coumarins [179].

On the other hand, several studies have been carried out on the synthesis of coumarins to produce coumarin derivatives with improved anticancer effects. Among them, synthetic scopoletin derivatives showed the greatest effects (IC50 < 2 µM) in MCF-7 and MDA-MB 231 cells (human breast adenocarcinoma cell line) as well as in HT29 cells (human colorectal adenocarcinoma cell line). The relationship between the increase in Log P value and the increase in cytotoxic activity was established in this study. Cell cycle arrest was also suggested as the anticancer mechanism of synthetic scopoletin derivatives [180]. 

Zhang et al. synthesized novel anticancer analogs of geiparvarin using a bioisosteric transformation method. In their study, it was also shown that adding electron-withdrawing substituents to the benzene rings, such as 7-((1-(4-fluorobenzyl)-1H-1,2,3-triazol-4-yl)methoxy)-4H-chromen-4-one, increased their cytotoxic effects in a human hepatoma cell line (QGY-7701, IC50 = 14.37 ± 9.93) and a colon carcinoma cell line (SW480, IC50 = 11.18 ± 2.16) compared with geiparvarin (IC50 = 17.68 ± 0.40 and 20.34 ± 0.75, respectively) [181]. 

In addition to the anticancer activity, coumarins have various pharmacological properties, including anti-HIV, antihypertensive, analgesic, antihyperlipidemic, anti-inflammatory and anticoagulant effects [46,182]. Conversion of prothrombin to thrombin is a crucial step in the coagulation cascade. Thrombin converts fibrinogen into fibrin which is a protein involved in blood clotting. Coumarins are competitive inhibitors of vitamin-K, which plays an important role in biosynthesis of prothrombin [183]. Additionally, hydroxy coumarin derivatives are multi-target agents that exhibit anticancer, antioxidant, hepatoprotective, antidiabetic, neuroprotective, anxiolytic, antidepressant, antibacterial and cardioprotective effects. One of these hydroxy coumarins, isofraxidin, has been revealed to attenuate several signaling pathways, such as NO, PGE2, TNF-α, IL-6, COX-2, iNOS, TLR4 leading to play an important role in the curation of several diseases [46]. 

## 3. Pharmacokinetics

Following oral intake, coumarins are rapidly absorbed from the gastrointestinal tract and spread throughout the body [184]. Coumarin and 7-hydroxycoumarin are both poorly soluble in water. However, both compounds have high partition coefficients. This indicates that coumarins can easily pass lipid double layers through passive diffusion, considering their nonpolar structure [149]. In clinical pharmacokinetic studies, it was determined that orally administered coumarin was completely absorbed from the GI system and intensely metabolized by the liver in the first pass, only 2–6% of it reached the systemic circulation [184].

The bioavailability of coumarine, which is the precursor of 7-hydroxycoumarin, is low and its half-life is short. In pharmacokinetic studies, it was determined that 35% of coumarin and 47% of 7-hydroxycoumarin bind to plasma proteins [184,185,186,187,188].

Coumarin is firstly metabolized by the specific cytochrome P-450-bound mono-oxygenase enzyme (CYP2A6) system in liver microsomes and converted into 7-hydroxycoumarin [189,190]. 7-Hydroxycoumarin is converted to glucuronide conjugate by phase-II conjugation [184,191,192]. The rapid excretion of coumarin and its metabolites occurs through urine [193,194,195,196]. 

Major adverse effect of coumarin as an anticoagulant agent is hemorrhage. The risk of bleeding is the most common side effect of long term oral anticoagulants [197,198]. Some factors such as increased patient age, female sex, 120 days or more of warfarin therapy during the year are related with the increased risk of bleeding [198]. Defects in the bone mineral metabolism is another side effect of coumarin anticoagulants related to their inhibitor effect on vitamin-K. Elderly patient’s especially postmenopausal females using long term anticoagulants tend to bone defects [197,199]. One of side effects of anticoagulants, such as coumarins, is full-thickness skin necrosis caused by thrombosis of arterioles and venues particularly in the skin of breasts, thighs and lower extremities. Usage of oral anticoagulants including coumarin derivatives in the gestation period constitutes a potential risk. Warfarin embryopathy which is characterized by nasal hypoplasia, upper respiratory obstruction and stippling in the region of the epiphyses of numerous bones is the most common complication of women taking warfarin who are pregnant between the sixth and twelfth weeks [197,198,199,200]. 

## 4. Toxicology

Coumarin was classified as a toxic substance by the FDA (Food and Drug Administration) in 1954 as it triggered liver tumor in rats [201] and the consumption of all foods containing coumarins is prohibited [149]. National Institute for Occupational Safety and Health (NIOSH) has named coumarin as a chemical carcinogen in tests performed on rodents. However, in many tests, such as Ames, micronucleus tests, coumarin and its metabolites have not been shown to be mutagenic [202]. The tumor-inducing effect of coumarin can be seen in sensitive species as organ damages and carcinomas. The uncertain effects of coumarin can be associated with the differentiation of its metabolism depending on the species. Therefore, extreme caution should be exercised in associating studies on both humans and animals [203]. In some studies, coumarin has been shown to cause acute, chronic and carcinogenic effects in rats and mice. In rats, it has been reported that it causes hepatic biochemical and morphological changes, causes a relative weight increase, causes liver necrosis in single-dose oral use and also increases plasma transaminase activities in DBA/2 strain mice [184]. In contrast, baboons, Syrian hamsters and some mouse strains resisted against acute coumarin-induced hepatotoxicity. Similarly, it has been determined that toxicity from in vitro coumarin varies by species. Studies on human and cynomolgus monkey liver fragments and/or hepatocytes have identified a relative resistance to coumarin toxicity, and this was associated with coumarin 7-hydroxylation, which is the main route of coumarin metabolism. It has been reported that coumarin-induced hepatotoxicity in rats can be attributed to the excretion of coumarin metabolites in bile. Low exposure to coumarins in dietary and cosmetic products is not expected to show hepatoxicity even in people with insufficient 7-hydroxylase activity [184,191].

## 5. Conclusions

The widespread distribution and various bioactivity of coumarins have led scientists to carry out research involving this ring system for decades. Coumarins have many biological activities, including disease prevention, growth modulation and antioxidant properties. It has been shown in scientific studies that these compounds show antitumor effects depending on their effects on immune regulation, cell growth and differentiation. It is extremely important to identify new, effective and less side effect anticancer products based on traditional medicines. Synthesis of coumarin and its derivatives is possible thanks to a number of innovative techniques, including the Pechmann, Claisen, Perkin, Knoevenagel and Wittig reactions. Although there are important limitations in use of most natural coumarins due to their hepatotoxic effect, relatively safe analogues with higher potency and thus better therapeutic index have been obtained by molecular manipulations. In the structure–activity studies on coumarins, significant positive results were obtained in anticancer activity screening with the addition of substituents at different positions of the coumarin core. Therefore, the development of new anticancer molecules by attaching appropriate functional groups to different positions around the coumarin core is an important research area.

However, significantly positive results were obtained in anticancer effect studies for various cancer types by targeting natural and synthetic coumarins to specific signaling pathways. Coumarin and coumarin-derived compounds are a potential source of anti-cancer drugs that need further researches, and it is obvious that they will be an important group in the development of new anticancer drugs.

## Figures and Tables

**Table 1:** Risk Factors in The Development of Breast Cancer.

*Demographic Features*	Gender, Age, Race/Ethnicity
**Reproductive story**	The age of menarche, number of birth, first full-term pregnancy age, menopause age, lactation, infertility, miscarriage
**Familial/genetic factors**	Family history, known or suspected BRCA1/2, p53, PTEN, or other gene mutations related to breast cancer risk
**Environmental factors**	Radiotherapy to the thorax before 30 years of age, hormone replacement therapy, alcohol use, socioeconomic level, etc.
**Other factors**	Personal history of breast cancer, number of breast biopsies, atypical hyperplasia or lobular carcinoma in situ, dense breast structure, body mass index (BMI)

**Table 2:** Risk factors in the development of malignant melanoma.

**Physical conditions**	Light skin color, blond or red hair, light eyes, freckles, or skin burns easily in the sun Sunburn history (formation of one or more severe, vesicle-forming sunburn) Having many or unusual moles
**Familial/genetic factors**	Family history of melanoma People diagnosed with melanoma in close relatives such as parents, children or siblings
**Environmental factors**	Life close to the equator or at high altitude Exposure to UV rays from the sun or solarium
**Weakened immune system**	People with weakened immunity (e.g., those with organ transplants)
